# Etiopathogenesis and Diagnostic Strategies in Autoimmune Hepatitis

**DOI:** 10.3390/diagnostics11081418

**Published:** 2021-08-05

**Authors:** Weronika Domerecka, Anna Kowalska-Kępczyńska, Agata Michalak, Iwona Homa-Mlak, Radosław Mlak, Halina Cichoż-Lach, Teresa Małecka-Massalska

**Affiliations:** 1 Chair and Department of Human Physiology, Medical University of Lublin, 20-080 Lublin, Poland; iwona.homa@wp.pl (I.H.-M.); radoslaw.mlak@gmail.com (R.M.); teresamaleckamassalska@umlub.pl (T.M.-M.); 2 Department of Biochemical Diagnostics, Chair of Laboratory Diagnostics, Medical University of Lublin, 20-081 Lublin, Poland; anna.kowalska-kepczynska@umlub.pl; 3 Department of Gastroenterology with Endoscopy Unit, Medical University of Lublin, 20-090 Lublin, Poland; lady.agatamichalak@gmail.com (A.M.); halina.lach@umlub.pl (H.C.-L.)

**Keywords:** autoimmune hepatitis, autoantibodies, LDG, NETs

## Abstract

Autoimmune hepatitis (AIH) is a chronic liver disease with the incidence of 10 to 17 per 100,000 people in Europe. It affects people of any age, but most often occurs in the 40–60 age group. The clinical picture is varied, from asymptomatic to severe acute hepatitis or liver failure. The disease onset is probably associated with the impaired function of T lymphocytes, the development of molecular mimicry, intestinal dysbiosis, or infiltration with low density neutrophils, which, alongside autoantibodies (i.e., ANA, ASMA), implicate the formation of neutrophil extracellular traps (NETs), as a component of the disease process, and mediate the inappropriate immune response. AIH is characterized with an increased activity of aminotransferases, elevated concentration of serum immunoglobulin G, the presence of circulating autoantibodies and liver inflammation. The result of the histological examination of the liver and the presence of autoantibodies, although not pathognomonic, still remain a distinguishing feature. The diagnosis of AIH determines lifelong treatment in most patients. The treatment is implemented to prevent the development of cirrhosis and end-stage liver failure. This work focuses mainly on the etiopathogenesis and diagnosis of AIH.

## 1. Introduction

Autoimmune hepatitis (AIH) is a complex immune disease of the liver [1,2]. It was first described in 1951 [3] as chronic hepatitis, occurring in young women with hypergammaglobulinemia, without cirrhosis, and responding well to adrenocorticotropic (ACTH) therapy [4].

AIH may be asymptomatic or manifest itself in various forms, ranging from subclinical disease to acute and end-stage liver failure [5]. Specific diagnostic criteria and classification systems have been established, including the presence of anti-nuclear antibodies (ANA), anti-smooth muscle antibodies (SMA), liver/kidney microsome type 1 antibodies (anti-LKM1), and anti-soluble liver antigen/liver pancreas (anti-SLA), immunoglobulin G (IgG), exclusion of viral markers (IgM antibody to hepatitis A virus (IgM anti-HAV), hepatitis B surface antigen (HBsAg), hepatitis B virus DNA (HBV DNA) and hepatitis C virus RNA (HCV RNA)) as well as the histological picture of the liver tissue [6]. According to the antibody profile, AIH can be divided into several subtypes: type 1, type 2, type 3 AIH and cryptogenic hepatitis [7]. The mainstays of AIH therapy are corticosteroids and immunosuppression. There are also therapeutic methods involving biological drugs [8], as well as cellular therapies [9]. Hepatic transplantation is a life-saving procedure in patients with acute liver failure caused by acute form of AIH, or in patients with chronic liver failure and in the case of hepatocellular carcinoma. Disease recurrence after hepatic transplantation is reported in 10–50% of patients, and the onset of de novo AIH has been described in both pediatric and adult liver transplant recipients [10].

## 2. Epidemiology and Classification

The incidence and characteristics of AIH differ in various geographic regions. AIH is a chronic disease with the incidence of 10 to 17 per 100,000 people in Europe, 17 cases per 100,000 in Norway and 31 cases per 100,000 in the US. The disease is considerably less frequent in Japan. A trend towards increased prevalence has been observed all over the world [11,12,13]. AIH was originally described in adolescent women. It is now known that it can occur in both genders, although it is more prevalent in women of all ages (70–80% of cases) all over the world and in all ethnic groups [7,14]. AIH is a multi-organ disease. AIH may coexist with other autoimmune diseases. They include hypothyroidism, ulcerative colitis, type 1 diabetes mellitus, rheumatoid arthritis, coeliac disease [7,14].

Epidemiological data show that 40–50% of cases are characterized by an acute onset of the disease, mainly in children [15], while 25% of patients already present with cirrhosis at the time of diagnosis. This indicates a long asymptomatic course of the disease leading to chronic or acute liver failure [16]. The course of AIH is, therefore, not always benign. Patients presenting with untreated severe disease are at high risk of death: 40% die within 6 months of diagnosis [12].

Currently, four types of AIH are distinguished. Type 1 AIH-more common in adults (45–70 years old), although it may also occur in younger people (10–20 years old). It is characterized by a mild course, the presence of antibodies (ANA and/or ASMA), predominantly against F-actin, and hypergammaglobulinemia. Type 2 AIH is characteristic for children and young adults (however, it is the most prevalent in children aged 2–14 years). It has a rapid course with a frequent presence of anti-LKM antibodies, among which there are three types: anti-LKM1, anti-LKM2, anti-LKM3.

Out of the three types of these antibodies (anti-LKM1, anti-LKM2 and anti-LKM3), anti-LKM1 are the most typical for type 2 AIH. Their presence can also be observed in the case of HCV infection. Other antibodies that are often detected in type 2 AIH are anti-SLA/LP antibodies and LC-1 (anti-liver cytosol antibodies). The clinical course of type 3 AIH is similar to type 1. It is characterized by the presence of anti-SLA/LP antibodies. Adult women aged 30–50 years constitute 90% of cases [17]. In addition, this type is associated with cryptogenic hepatitis with no antibodies found in the patient’s serum. Table 1 shows the diversification of respective types of AIH according to the occurrence of particular autoantibodies [12,18].

Anti-smooth muscle antibodies (ASMA), anti-nuclear antibodies (ANA), liver/kidney microsome antibodies (anti-LKM), anti-soluble liver antigen/liver pancreas antibodies (anti-SLA/LP), anti-liver cytosol antibodies (anti-LC1).

## 3. Etiology

**Figure 1:**
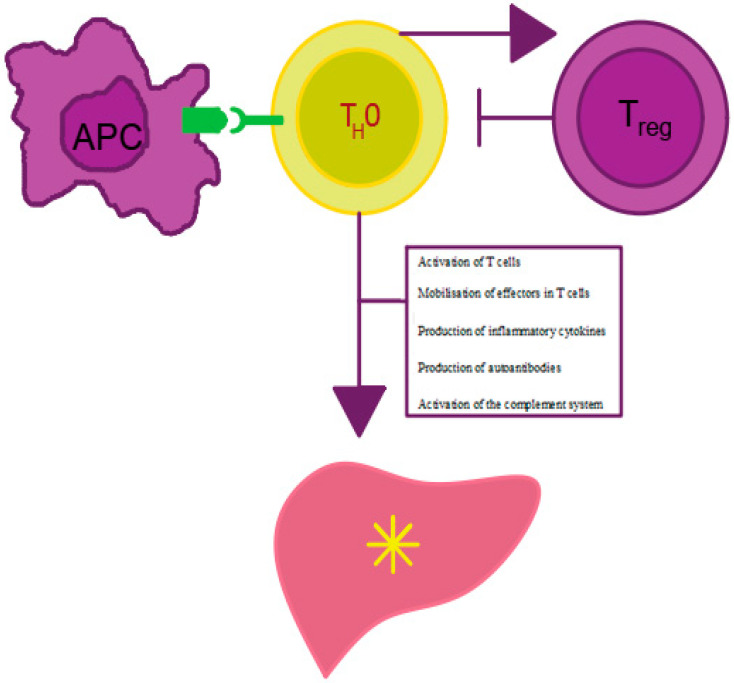
Mechanism of AIH development. APC—antigen presenting cell, Treg—regulatory T cell, Th0—T helper cell. Own elaboration based on [16]. Antigen presentation by APC cells to Th0 lymphocytes leads to effector mobilization on Treg cells and proinflammatory cytokine production. The cytokines stimulate antibody maturation and production by B lymphocytes and inhibit Treg lymphocyte activity. The decrease in the number of Treg lymphocytes leads to the impairment of tolerance to autoantigens, which, in turn, results in the initiation and persistence of autoimmune liver damage.

AIH etiology is not fully understood. The most important role may be attributed to genetic, environmental, toxic and infective factors, including hepatitis A virus (HAV) [21,22], hepatitis C virus (HCV) [23,24], hepatitis E virus (HEV) [25], measles morbillivirus [26], Epstein–Barr virus (EBV) [16] and herpes simplex virus (HSV) [27] as well as medicines [28,29]. It has been proven that drug metabolites may stimulate the production of antibodies against liver cells. Thus, the stimulation of the immune system may occur years before the occurrence of the first disease symptoms [30]. It is thought that the immune response directed towards hepatic autoantigens initiates and perpetuates liver damage. It has been demonstrated that there is an association between AIH etiology and changes within the human leukocyte antigen region (*HLA*) as well as major histocompatibility complex (MHC), located on the short arm of chromosome 6, which is involved in the presentation of antigen peptides to T lymphocytes and in the initiation of adaptive immune response [31,32] (Figure 1). The Genome of the Netherlands Project [33] indicated *HLA*-*DRB1* * 0301 and DRB1* 0401 as genotypes associated with the susceptibility to AIH, while *SH2B3* and *CARD10* (genes in a region other than *HLA*) turned out to be significantly associated with AIH. A study by Cheh et al. [34] also suggests that allele (HLA)-DRB1 * 16:02) is associated with the pathomechanism of many autoimmune diseases such as systemic lupus erythematosus, anti-N-methyl-d-aspartate receptor (NMDAR) encephalitis, Graves’ disease, myasthenia gravis, neuromyelitis optica and antibody-associated systemic vasculitis with microscopic polyangiitis (AASV-MPA) however, it is not associated with type 1 AIH, multiple sclerosis or rheumatoid arthritis.

### 3.1. Molecular Mimicry and Intestinal Dysbiosis in Autoimmune Hepatitis (AIH)

Molecular mimicry is one of the potential mechanisms leading to AIH in patients with increased genetic susceptibility. It works by inducing an immune response to exogenous pathogens that stems the production of antibodies that cross-react with liver autoantigens. This is due to their structural similarity to the antigens of pathogenic microorganisms of a similar structure [18,35,36,37,38]. Molecular mimicry is, therefore, based on the structural and sometimes also functional similarity between antigens of a microorganism and human antigens. An example of such a phenomenon in AIH is the homology of the biochemical structure of HCV, CMV (*Cytomegalovirus*) and HSV-1 viruses and the cytochrome P450 IID6 [39,40,41,42]. It has been shown that this antigen, as well as the short linear amino acid sequences of the CYP IA2 and CYP IIC11 proteins present in liver microsomes, can be recognized as microbial antigens by the serum antibodies present in AIH patients. The key role is attributed to the CYP IID6 molecule being the main antigen of anti-LKM-1 autoantibodies, which are characteristic for type 2 AIH [43].

Molecular mimicry is also recognized as a possible key element of microbiome-related autoimmunity. The gastrointestinal microflora plays an important role in shaping the intestinal and systemic immune response [44,45,46,47,48,49]. Its composition depends on gender, ethnicity, age, diet, and socioeconomic status [50,51,52,53]. Bacterial components of the intestinal microbiome can activate Toll-like receptors (TLRs) [51], contributing to the formation of inflammasomes, i.e., multiparticulate protein complexes that mediate the inflammatory response [54,55,56,57], stimulate the systemic immune response [58,59] and activate the intestinal immune cells that migrate to the peripheral lymphoid tissue [60,61]. Changes in the microbial composition of the intestine (dysbiosis) have already been associated with many diseases, such as type 1 diabetes [62], multiple sclerosis [63], inflammatory bowel disease [64,65], NAFLD (non-alcoholic fatty liver disease) [56], PBC (primary biliary cholangitis) [66,67], PSC (primary sclerosing cholangitis) [67,68] and AIH [68,69].

Patients with AIH demonstrate deficiencies in the zonula occludens 1 (ZO-1) and occludin structural proteins, which maintain the integrity of the mucosal barrier of the gastrointestinal tract [70]. In addition, they also show increased plasma levels of gut derived lipopolysaccharides (LPS) and a reduced amount of anaerobic bacteria [70,71]. Changes in the microbiome composition can lead to increased intestinal permeability, which in turn facilitates the passage of bacteria into the portal circulation [72,73,74]. Changes in the intestinal microflora have recently been described based on studies using an experimental humanized mouse model of AIH [69]. Also, a study by Wei et al. demonstrated changes in the composition and function of the intestinal microbiome in patients with AIH, which indicates the possibility of using the composition of the intestinal microbiota as a non-invasive biomarker for the assessment of disease activity [75].

### 3.2. Low-Density Granulocytes (LDG) and Neutrophil Extracellular Traps (NETs) in AIH

There is ample evidence for the role of neutrophils and their distinct LDG (low-density granulocytes) subset in the development of AIH [76]. LDGs are neutrophils which, after separation using density gradient centrifugation, remain in the peripheral blood mononuclear cell (PBMC) fraction [77]. When they were first described, it was noted that they may be present in many rheumatic diseases, such as systemic lupus erythematosus (SLE) [78] and rheumatoid arthritis (RA) [79]. More recent studies also describe LDG in diseases such as asthma [80], tuberculosis [81], psoriasis [82], animal models of viral infection [83,84], arthritis [85], in people with cancer [86], sepsis [87], HIV [88,89], and various autoimmune diseases including AIH [76].

Similarly, to normal-density granulocytes (NDG), LDG neutrophils strongly express LDG-specific surface markers (CD10, CD15 and CD16), but, in contrast to NDG, their nucleus is immature [90,91]. LDGs exhibit features of reactive cells. When activated, they can damage endothelial cells and release large amounts of tumor necrosis factors (TNF) and type I and II interferons (IFNs) [90,92,93]. Activated by pathogenic microorganisms or pro-inflammatory cytokines, LDGs can undergo spontaneous death (i.e., the release of neutrophil extracellular traps (NETs), which is also known as NETosis). NETs are composed of DNA, granules (including proteolytic enzymes) and the contents of the cell nucleus, which are released into the extracellular space [94,95]. Trapped in the chromatin NETs, pathogens are exposed to various substances, incl. cationic serine protease (proteinase 3, cathepsin G, neutrophil elastase (NE)), myeloperoxidase (MPO), bactericidal/permeability-increasing protein (BPI protein) with bactericidal permeability enhancing properties, lactoferrin, gelatin B, cathelicidin (LL-37 or cathelicidin antimicrobial peptide CAP-18) as well as histone proteins (core histones and linker histones H1) and tryptase [96].

The released substances destroy pathogenic microorganisms, contributing to inflammation and tissue damage [97,98].

The involvement of NETs in AIH as a trigger mechanism has not yet been studied in detail. Probably the LDG infiltration, which is observed at the early stage of AIH, alongside the dominant autoantibodies, i.e., ANA or ASMA, implies the formation of NETs as a component of the disease process and influences an abnormal immune response. These studies suggest that the imbalance between the NET formation process and their degradation may be related to the development of autoimmune diseases. Neutrophils, interleukin-8, ANCA (anti-neutrophil cytoplasmic antibodies), and other inflammatory molecules are believed to play a key role in inducing NETs. Long-term exposure of organs to NETs is associated with the intensification of the autoimmune process and thus a greater risk of their damage [98].

However, both the level and the clinical significance of LDG associated with AIH are not yet understood. Nevertheless, growing evidence indicates that uncontrolled or excess production of NETs is associated with exacerbation of inflammation and the development of autoimmunity in AIH. Nevertheless, research related to NETs may be helpful in elucidating the mechanism of AIH development and in the elaboration of novel diagnostic and therapeutic strategies.

## 4. Clinical Symptoms and Diagnostics of AIH

AIH is asymptomatic for a long time. The most common clinical symptoms of advanced forms of AIH include ascites (91% of patients), progressive jaundice (69–88% of patients), anorexia, asymptomatic hepatosplenomegaly (50% of patients) and abdominal pain (over 50% of patients). 20% of patients with AIH develop epistaxis, acne, fever and tender hepatomegaly, which often is the only clinical symptom. Regardless of the stage of the disease (exacerbation or remission), patients often complain of bothersome weariness and fatigue. The presence of these symptoms should be taken into account when diagnosing chronic fatigue syndrome.

The diagnosis of AIH is difficult, even in the case of an acute relapse [99]; the laboratory markers do not always facilitate a clear diagnosis. Moreover, diagnostic difficulties often concern a group of patients with features of already developed chronic liver damage due to AIH. In such case, a detailed differential diagnosis should be carried out in order to establish the pathogenesis of these disorders. It is essential to eliminate the potential toxic damage to hepatocytes (mainly alcohol abuse) and the possible infection with hepatotropic viruses (e.g., HBV, HCV, CMV) as well as biliary pathology. Thereafter, it is justified to start the diagnostics towards autoimmune liver diseases. In clinical practice, the following three most common disease entities are usually considered: AIH, PBC and PSC. The clinical picture of AIH commonly differs from PBC and PSC, nevertheless, the above diseases may occur concomitantly, which may also constitute a real diagnostic challenge in some cases. However, AIH usually manifests itself insidiously, beginning with intensifying symptoms of chronic liver disease, and patients can sometimes be diagnosed after accidental detection of abnormal liver function tests [100]. Moreover, other autoimmune diseases such as PBC and PSC [101], as well as systemic sclerosis [102] are also encountered in patients with AIH.

**Figure 2:**
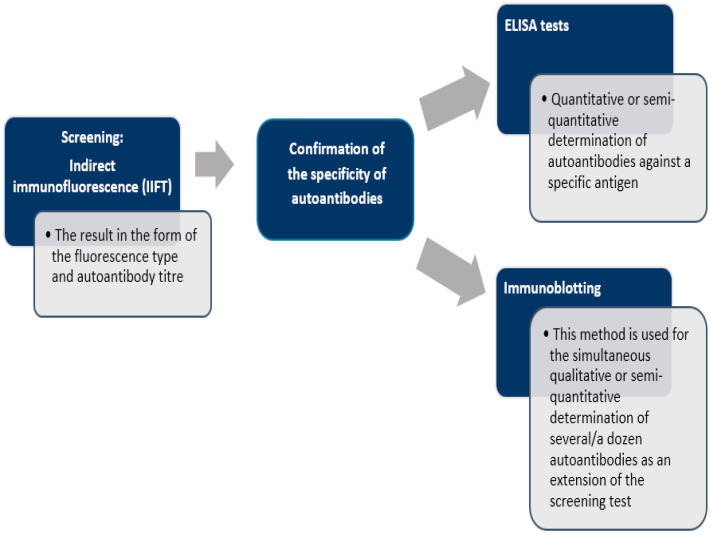
Scheme of laboratory diagnosis of autoimmune liver disease [19,105].

There is no single test that allows definitive diagnosis of AIH. The diagnosis is based solely on a combination of clinical, serological, biochemical, and histological findings. One of the most important diagnostic criteria for autoimmune hepatitis is the presence of serum autoantibodies, which can be detected by such methods as: indirect immunofluorescence technique (IIFT) [103], enzyme-linked immunosorbent assay (ELISA) [104] or immunoblotting [105] (Figure 2). Laboratory tests demonstrate hypergammaglobulinemia and a selective increase in IgG, sometimes a slight increase in IgM, elevated transaminase activity, especially of alanine aminotransferase (ALT) and aspartate aminotransferase (AST), usually 5-fold above normal values. Sometimes, in advanced stages of the disease, increased bilirubin and hypoalbuminemia are observed. The levels of cholestasis markers—alkaline phosphatase (ALP) and gamma-glutamyltransferase (GGTP)—are usually normal or slightly elevated. Other causes of hepatitis should be carefully ruled out, especially alcohol, drugs or viruses.

## 5. Autoantibodies

The main autoantibodies in AIH include: ASMA, ANA, anti-SLA/LP, anti-LKM and anti-LC1. The first diagnostic criteria were established in 1992 by the Autoimmune Hepatitis Group [106]. They were then revised in 1999 [107]. However, the revised criteria included complex and insufficiently validated parameters of questionable value, which were mainly developed to allow comparisons of studies from different centers. Consequently, simplified criteria were proposed that included 4 instead of 12 diagnostic parameters to facilitate wider application in routine clinical practice. These criteria, constituting the basis for result calculation, include the quantification of autoantibodies (ANA, anti-SMA, anti-LKM-1 antibody titers), immunoglobulin G, and the assessment of liver histology (evidence of hepatitis, lymphoplasmatic infiltration) [7]. The potential diagnosis is considered when the score equals 6. The score equal to or greater than 7 indicated definitive diagnosis of AIH. The simplified criteria are presented in Table 2 [6,7,108,109].

The measurement of autoantibodies, which has been included in all scoring systems, is a key step in both the diagnosis and classification of AIH, and should be performed in all patients suspected of this disease [110].

**ASMA, or anti-SMA.** They react with the antigens of the muscle structures: F-actin and non-active components of the cellular cytoplasm. Their high and persistent titer (>1:1000) is one of the criteria for the diagnosis of AIH-1. They occur in 87% (40–90%) of patients with AIH-1 (in 33% as the only ones, in 54% in combination with ANA). It should be kept in mind that low ASMA titers are found in viral drug-induced hepatitis, alcoholic cirrhosis, biliary obstruction and visceral lupus [19].

**Anti-SLA/LP antibodies** are a highly specific marker of autoimmune hepatitis and indicate a severe course of the disease. The diagnostic value of anti-SLA/LP is close to 100%—any positive result is very likely to indicate type 1 AIH, provided that the appropriate clinical symptoms are present. Unlike other autoantibodies, anti-SLA/LP antibodies are highly specific for AIH and have not been reported in other diseases. Anti-SLA/LP can be present in type 1 AIH together with other autoantibodies or as a sole marker [111].

**Anti-LKM-1 antibodies** can occur in both type 2 AIH and HCV infections. The prevalence of AIH in adults is about 1%, being more common in children. Other types of anti-LKM antibody are not associated with autoimmune liver diseases, anti-LKM-2 are present in patients with drug-induced lesions, and anti-LKM-3 are present in 10–15% of patients with HBV/HDV [111].

**Anti-LC1 antibodies** are associated with a more severe form of the disease-type 2 AIH. The prevalence of anti-LC1 antibodies is 1–2%; they are more common in patients with anti-LKM-1 without HCV infection. They are the only indicator of the disease in 14% of patients. They can be detected by indirect immunofluorescence method, however, due to the frequent simultaneous presence of anti-LKM-1 autoantibodies, which react in the same areas of the liver tissue, they can easily get overlooked. For this reason, it is recommended to perform a monospecific test. ELISA or immunoblotting are used to confirm their presence. The target antigen for anti-LC1 antibodies is formimidoyltransferase cyclodeaminase (FTCD) and this recombinant protein is the antigen used in the ELISA and immunoblotting methods [112].

### 5.1. Histological Findings in Liver Biopsy Specimens Obtained from AIH Patients

According to the American Association for the Study of Liver Diseases (AASLD) [113] and European Association for the Study of the Liver (EASL) [114] guidelines, liver biopsy still remains a recommended gold standard in the diagnosis of AIH and in the evaluation of liver fibrosis stage during the disease. Moreover, histological demonstration of hepatitis constitutes a core component of commonly used diagnostic scores in AIH. Simultaneously, liver biopsy makes it possible to exclude other potential causative factors of existing liver disorder and finally to establish the further management of AIH patients. It is also the only way to confirm the presence of the disease in case of the lack of serological antibodies (up to 20% cases of AIH) [115]. The key histological disturbance in the pathological appearance of AIH is reflected by chronic active hepatitis, which includes marked portal inflammation, interface hepatitis and lobular hepatitis with varying severity [16]. The character of histological findings depends on the stage of disease; however, a hepatic pattern of injury is typical [116,117]. Hepatocellular damage together with parenchymal necrosis or apoptosis are distinctive for lobular hepatitis and might be found within lobular zones and periportal areas. Furthermore, damaged limiting plates form an interface between lobular hepatitis and portal hepatitis. A migration of lymphocytes and plasma cells from the portal tract into the area of periportal parenchyma occurs and this forms interface hepatitis (piecemeal necrosis)-a typical hallmark of chronic active hepatitis regardless of its etiology and directly linked to the disease progression. Thus, an active phase of AIH is reflected by the presence of interface hepatitis. The spectrum of lobular hepatitis includes single cell necrosis, spotty necrosis, focal necrosis, confluent necrosis (bridging/zonal necrosis) and submassive/massive necrotic lesions with reference to the degree of hepatocellular damage and location of affected lobules. Emperipolesis (the phenomenon of engulfment of lymphocytes by hepatocytes) might be observed in zones of interface activity even in approximately 70% of AIH cases. However, emperipolesis does not belong to pathognomonic characteristics of AIH and is also described in patients with PBC, DILI (drug-induced liver injury) or chronic viral hepatitis. The engulfed cells are mainly represented by CD8+ T cells and CD56+ NK cells (ang. natural killer T) and rarely plasma cells. In the majority of cases, the presence of emperipolesis in AIH is accompanied by coexisting high activity of transaminases in serum and necroinflammatory lesions in liver parenchyma including confluent necrosis of centrilobular area (centrilobular necrosis) [118,119]. Additionally, the presence of hepatocyte rosette-like formation due to the thickening of liver cell plates during regeneration is also common in AIH. Prominent plasma cells are often in the portal inflammatory infiltrate, nevertheless one-third of biopsies obtained from AIH patients will contain few or no plasma cells. Of note, severe portal inflammation associated with AIH might be accompanied by bile ducts damage (typical in the course of PBC or PSC). Moreover, perivenular necroinflammatory activity, characterized by prominent mononuclear inflammation and hepatocellular necrosis surrounding terminal hepatic venules, can be found especially in the early phase of AIH. Simultaneously, the regeneration of hepatocytes occurs closely to necrotic regions. These regenerating cells organize into clusters of small monomorphic hepatocytes with clear cytoplasm giving a “cobblestone” appearance in biopsy specimens from AIH patients. In this cobblestone space, the trabeculae of hepatocytes tend to be thick and hepatic rosette formation is often seen as the consequence of central lumen formation. The fibrosis stage at the time of diagnosis varies from no fibrotic changes, through minimal fibrosis and finally to established liver cirrhosis. The already developed cirrhotic stage of AIH might have no pathognomonic histopathological features (so-called burnt-out disease) and the diagnosis of existing AIH can be based only on clinical presentation and serologic data [120,121,122].

### 5.2. AIH Treatment—Known Players and Future Perspectives

**Figure 3:**
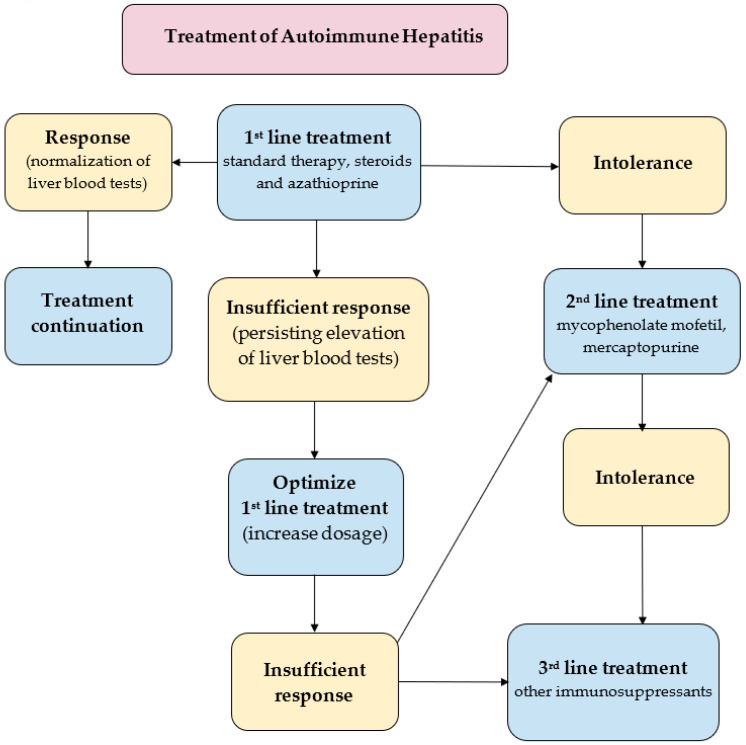
Treatment strategies in AIH [113,114,125,127].

A key goal in the treatment of already diagnosed AIH is to control hepatic inflammation to achieve clinical, biochemical and histopathological remission of the disease, (Figure 3). The most favorable profile of biochemical response to the treatment is the normalization of the serum AST, ALT, and IgG levels. Histopathological remission results from biochemical response. Of note, sustaining biochemical remission for a long time (>1 year from the application of treatment) constitutes a surrogate for satisfying overall long-term survival [13]. The first-line paradigm is based on corticosteroids in monotherapy or in combination with azathioprine (AZA). The 2019 AASLD guidelines propose either prednisone monotherapy (40–60 mg/day) or a combination of prednisone (20–40 mg/day) or budesonide (9 mg/day) and AZA (50–100 mg/day) [113]. The 2015 EASL guidelines recommend 0.5–1 mg/kg/day predniso(lo)ne as the initial treatment, followed by a 50 mg/day AZA add-on [114]. The AASLD also suggests a period of a 2-week observation before the initiation of AZA in order to confirm the patient’s steroid responsiveness and to evaluate the status of thiopurine-S methyltransferase (TPMT) to avoid AZA-induced hepatitis. TPMT metabolizes thiopurines (e.g., AZA) and single nucleotide polymorphisms of its genes can result in the loss of enzymatic activity. This disorder predisposes patients (especially European and African descendants) to the development of thiopurine-related toxicity [123]. To evaluate the relevance of the starting dose of predniso(lo)ne, a retrospective observational study was performed in nine sites located in five European countries. This demonstrated that an initial low dose of AZA in the majority of patients (>85%) significantly decreased the unnecessary exposure to predniso(lo)ne in more than 85% of patients. Furthermore, budesonide has been proved to cause less systemic side effects, due to a 90% first-pass hepatic clearance rate [124]. The AASLD even demonstrated a higher rate of biochemical remission in the budesonide + AZA group compared to the prednisone + AZA group (odds ratio, 2.19; 95% CI, 1.30–3.67). Thus, the AASLD suggests budesonide in combination with AZA as a first-line therapy for pediatric and adult AIH patients without cirrhosis or acute severe AIH. Budesonide is contraindicated in cirrhotic patients because of portosystemic shunting which may reduce the drug’s efficacy. The combination of AZA with either predniso(lo)ne or budesonide is now believed to be the most standard first-line therapy of AIH in western countries [13,125]. As corticosteroids are still the mainstay of the first-line treatment of AIH, limiting corticosteroids-related osteoporosis in patients with risk factors is required during treatment to promote bone preservation [126]. In patients with refractoriness, incomplete biochemical response and intolerance to first-line treatment, mycophenolate mofetil (MMF), calcineurin inhibitors (cyclosporin A, tacrolimus), mercaptopurine, and biologics (e.g., infliximab) are taken into consideration. The combination of MMF and prednisone appears to be the most widely suggested second-line treatment, leading to histological remission in 89% of the patients [127]. Another survey confirmed the effectiveness of MMF as a second-line therapy for AIH patients who have failed standard therapy; the rate of induction of biochemical remission was 60% [128]. A significant number of AIH patients will have to face spontaneous and asymptomatic exacerbation or recrudescence, reflected by an increased level of ALT (with or without elevation in IgG). Multiple relapses are known to be surrogates of worse outcomes [129]. On the other hand, a sustained biochemical remission of ≥2 years was suggested by the AASLD as the eligibility criterion for considering a treatment withdrawal. AIH with already decompensated cirrhosis in its course or acute liver failure requires liver transplantation (LT). According to the results of the prospective multicenter European Liver Transplant Registry (1998–2017), the general survival of patients after AIH-LT was reported to be comparable to that of patients after alcohol-related cirrhosis-LT, however, worse than in the case of primary biliary cholangitis-LT and primary sclerosing cholangitis-LT.69 The 5- and 10-year patient survival rates in the AIH group were 79.4% and 73.25%, respectively, with corresponding graft survival rates of 70.8% and 63.4% [130]. The future of the AIH treatment rests in the novel agents directly targeting the early stages of the inflammatory cascade responsible for the development of the disease. Anti-B cell-activating factor of the tumor necrosis factor family (BAFF) and anti-tumor necrosis factor (TNF) therapies, novel peptides, T regulatory cells, interleukin (IL)-1 or IL-6 blockade and Janus kinases are worth mentioning as potential players in this field. But there is still a great need for subsequent studies assessing their usefulness in AIH patients [131,132].

## 6. Summary

In recent decades, significant progress has been made in understanding the pathogenesis and diagnosis of AIH, but the exact cause of this disease’s development is still unknown. Human genome studies have identified the key predisposing HLA allelic variants associated with the development of AIH. In addition, an increasing number of studies are suggesting a significant role of NETs in exacerbating inflammation and the occurrence of autoimmune diseases, including AIH.

The currently used diagnostic systems are characterized with high sensitivity and specificity, but there is still no test that would quickly and easily contribute to the detection of this disease. However, thanks to the advancement of science, it is possible to explore the most unconventional etiologies, such as NETs, and the intestinal microbiome as factors contributing to the development of the disease. In the future, this knowledge can contribute to the implementation of new therapies and innovative diagnostic methods.

## Figures and Tables

**Table 1:** Autoimmune hepatitis (AIH) classification according to the presence of autoantibodies based on [12,19,20].

AIH Types		
Type	Antibody	Prevalence
1	ASMA	40–90%
	ANA	5–70%
	Anti-SLA/LP	10–30%
2	Anti-LKM-1	2–4%
	Anti-LC1	1–2%
3	Anti-SLA/LP	10–30%
Cryptogenic hepatitis	Absence of antibodies	

**Table 2:** Simplified criteria for AIH diagnostics of the International Autoimmune Hepatitis Group (IAHG) [7,12,18].

Parameter	Value	Score
ANA or ASMA	>1:40 >1:80	1 2
or LKM	>1:40	2
or SLA	Present	2
IgG	>ULN Over 10% above ULN	1 2
Histopathologic examination	Corresponding to AIH Typical for AIH	1 2
Hepatotropic viruses	Negative test result	2

ANA—anti-nuclear antibodies; ASMA—anti-smooth muscle antibodies; SLA—anti-soluble liver antigen; LKM—anti-liver/kidney microsome antibodies; IgG—immunoglobulin G; ULN—upper limit of normal.

## Data Availability

Not applicable.

## Abbreviations

AASLD	American Association for the Study of Liver Diseases
ACTH	Adrenocorticotropic Hormone
AIH	Autoimmune Hepatitis
AIH-LT	liver transplantation for autoimmune hepatitis
ALP	Alkaline Phosphatase
ALT	Alanine Aminotransferase
ANA	Anti-Nuclear Antibodies
ANCA	Anti-Neutrophil Cytoplasmic Antibodies
anti-LC1	Anti-Liver Cytosol antibodies
anti-LKM	Liver/Kidney Microsome antibodies
anti-SLA	Anti-Soluble Liver Antigen/Liver Pancreas
anti-SMA (ASMA)	Anti-Smooth Muscle Antibodies
AST	Aspartate Aminotransferase
AZA	Azathioprine
BAFF	B-cell activating factor
BPI protein	Bactericidal/Permeability-Increasing protein
CMV	Cytomegalovirus
DILI	Drug-Induced Liver Injury
DNA	Deoxyribonucleic Acid
EASL	European Association for the Study of the Liver
ELISA	Enzyme-Linked Immunosorbent Assay
FTCD	Formimidoyltransferase Cyclodeaminase
GGTP	Gamma-Glutamyltransferase
HAV	Hepatitis A Virus
HBsAg	Hepatitis B surface Antigen
HBV	Hepatitis B Virus
HCV	Hepatitis C Virus
HDV	Hepatitis D Virus
HIV	Human Immunodeficiency Virus
HLA	Human Leukocyte Antigens
HSV	Herpes Simplex Virus
IFN	Interferon
IgG	Immunoglobulin G
IIFT	Indirect Immunofluorescence Technique
LDGs	Low-Density Granulocytes
LPS	Lipopolysaccharides
MMF	Mycophenolate mofetil
MPO	Myeloperoxidase
NAFLD	Nonalcoholic Fatty Liver Disease
NDG	Normal-Density Granulocytes
NE	Neutrophil Elastase
NETs	Neutrophil Extracellular Traps
PBC	Primary Biliary Cholangitis
PBMC	Peripheral Blood Mononuclear Cell
PSC	Primary Sclerosing Cholangitis
RA	Rheumatoid Arthritis
SLE	Systemic Lupus Erythematosus
TLRs	Toll-like Receptors
TNF	Tumor Necrosis Factors
TPMT	Thiopurine-S methyltransferase
ULN	Upper Limit of Normal
ZO-1	Zonula Occludens 1
